# Application of Machine Learning and Mendelian Randomization Analysis to Identify the Cuproptosis-Related Biomarker and Its Related Regulation in Osteonecrosis of the Femoral Head

**DOI:** 10.2174/0113892029373131251017114954

**Published:** 2025-10-23

**Authors:** Linxiang Wang, Hongming Meng, Han Zhou, Zeyu Shou, Liangyan Chen, Xiaojing Huang, Zhibiao Bai, Chun Chen

**Affiliations:** 1 Department of Acupuncture, Massage, and Physical Therapy, The First Affiliated Hospital of Wenzhou Medical University, Wenzhou City, 325000, Zhejiang Province, China;; 2 Department of Orthopedics, The First Affiliated Hospital of Wenzhou Medical University, Wenzhou City, 325000, Zhejiang Province, China;; 3 Department of Orthopedics, Shaoxing Hospital of Traditional Chinese Medicine, Shaoxing TCM Hospital Affiliated to Zhejiang Chinese Medical University, Shaoxing, Zhejiang, China;; 4 Key Laboratory of Intelligent Treatment and Life Support for Critical Diseases of Zhejiang Province, Wenzhou, 325000, Zhejiang, China;; 5 Zhejiang Engineering Research Center for Hospital Emergency and Process Digitization, Wenzhou, 325000, Zhejiang, China

**Keywords:** Osteonecrosis of the femoral head, cuproptosis, SMR, immune infiltration, machine learning, biomarker

## Abstract

**Introduction:**

Osteonecrosis of the Femoral Head (ONFH) is one of the common refractory diseases. However, the role of cuproptosis in ONFH pathogenesis remains unexplored. This study aimed to investigate the potential relationship between cuproptosis and ONFH.

**Methods:**

ONFH-related datasets were obtained from the Gene Expression Omnibus (GEO) database, and cuproptosis-related genes in the GSE123568 dataset were identified through differential expression analysis. To further discover potential cuproptosis-related biomarkers, Least Absolute Shrinkage and Selection Operator (LASSO) regression analysis and Support Vector Machine (SVM) analysis were conducted. The Receiver Operating Characteristic (ROC) curve analysis was used to explore the diagnostic value of cuproptosis-related biomarkers. The summary Statistics-based Mendelian Randomization (SMR) algorithm was used to investigate the causal relationship between the related genes and ONFH. The immune infiltration analysis was conducted to assess the effect of immune cells on ONFH. Subsequently, the GSE74089 and GSE89587 datasets were used to validate gene expression levels and predict the lncRNA-miRNA-mRNA network. Finally, quantitative Real-Time Polymerase Chain Reaction (qRT-PCR) was employed to validate the expression of these genes.

**Results:**

The study showed that the upregulation of *PDHB*, a cuproptosis-related biomarker, may contribute to the development of ONFH. Additionally, immune cells were found to play a crucial role in ONFH, and PDHB showed a significant association with various immune cells. Furthermore, the study identified the existence of the *MIR22HG*/*let-7c-5p*/*PDHB* regulatory pathway, which may play a critical role in ONFH through cuproptosis.

**Discussion:**

This study discovered a cuproptosis-related regulating pathway, MIR22HG/let-7c-5p/PDHB. This can provide new insights into the treatment of ONFH. However, further experimental validation is needed.

**Conclusion:**

*PDHB*, identified as a cuproptosis-related biomarker, can induce ONFH through cuproptosis. *PDHB* also contributes to the pathogenesis and progression of ONFH by influencing immune cell function. This is most likely mediated through the regulatory interaction between *MIR22HG, let-7c-5p,* and *PDHB*.

## 1 INTRODUCTION

ONFH is a prevalent refractory disease in orthopedics and has a multifactorial etiology. Based on the current research, the primary risk factors for ONFH include trauma, corticosteroid use, alcohol consumption, hyperlipidemia, and blood metabolism abnormalities [1-3]. The precise pathogenic mechanism of ONFH remains elusive, and it may involve a complex interplay of environmental and genetic factors. Several theories regarding ONFH pathogenesis have been proposed, including lipid metabolism disorders, endothelial cell damage and hypercoagulable state, osteocyte apoptosis, autophagy and apoptosis, and oxidative stress [4-8]. Although multiple treatment modalities exist for ONFH, including pharmacological interventions, physical therapy, and surgical procedures, the rapid progression of the condition often necessitates surgical intervention as the optimal treatment for most patients; nevertheless, several issues require urgent attention [9-13]. Consequently, a comprehensive understanding of the etiology, pathogenetic mechanisms, and treatment approaches for ONFH is crucial for advancing research in this field.

Copper is an essential element for human health and functions as a cofactor for crucial enzymes. An imbalance in copper levels can lead to cell death. Recent research has identified a novel form of cell death, termed cuproptosis, which primarily occurs through the Tricarboxylic Acid (TCA) cycle [14]. Current evidence suggests that copper-induced mortality plays a remarkable role in various diseases, including tumors, idiopathic pulmonary fibrosis, alcoholic liver disease, and Osteoarthritis (OA) [15-18]. Gang Li found that cuproptosis is associated with multiple cancer hallmarks, the immune microenvironment, drug resistance, and patient survival; this finding highlights the importance of cuproptosis in human cancers [19]. Shuying Liu revealed that 11 cuproptosis-related genes regulate periodontitis development by influencing immune cells [20]. Furthermore, copper is vital for bone metabolism, and its metabolic disorders can lead to various bone diseases [21]. Baochuang Qi *et al.* showed that cuproptosis was closely associated with steroid-induced ONFH [22]. However, the regulatory molecular and genetic mechanisms underlying cuproptosis in ONFH remain largely unexplored.

In recent years, because of the increasing availability of high-throughput technologies, bioinformatics has gained prominence as an essential tool across various biological fields, including the diagnosis, prevention, and monitoring of ONFH [23-25]. Concurrently, Genome-Wide Association Studies (GWAS) have identified critical genetic variants associated with diverse complex traits, including both qualitative traits (such as cancer, plasma proteins, and metabolites) and quantitative traits (such as height) [26, 27]. Transcriptome-Wide Association Studies (TWAS) have also revealed numerous important gene-trait associations [28, 29]. Furthermore, Mendelian Randomization (MR) is widely utilized in medical research studies using GWAS data, such as studies on tumors, OA, ankylosing spondylitis, and ONFH [30-33]. Studies have also explored the causal relationship between the gut microbiota, immune cells, and osteonecrosis [34]. Therefore, the current research aimed to conduct comprehensive bioinformatics and MR analyses using publicly available datasets to identify gene features related to cuproptosis in ONFH, thereby enhancing our understanding of cuproptosis-related mechanisms in ONFH. This research may elucidate key pathogenic mechanisms and potential therapeutic targets for ONFH, potentially guiding future experimental studies.

## 2 MATERIALS AND METHODS

### 2.1 Data Processing

The soft matrix files of the GSE123568, GSE74089, and GSE89587 datasets were acquired from the GEO database using the “GEOquery” package (version 2.54.1) [35]. The GSE123568 dataset was utilized for differential analysis to identify cuproptosis-related Differentially Expressed Genes (DEGs). The GSE74089 dataset was used to confirm the expression of target mRNAs and lncRNAs. The GSE89587 dataset was used to validate the expression of the target miRNA. Cuproptosis-related genes were obtained from previously published literature and the FerrDb database (http://www.zhounan.org/ferrdb/current/) [14, 36].

### 2.2 Identification of Cuproptosis-related DEGs in ONFH

The datasets were downloaded using the GEOquery package (version 2.54.1) from the GEO database. Probes corresponding to multiple molecules were eliminated. Additionally, when more than one probe is found to correspond to the same molecule, only the probe with the highest signal value is retained. Subsequently, the data were normalized again by the normalizeBetweenArrays function of the limma package (version 3.42.2) [37]. To verify the efficacy of the normalization process, sample normalization was assessed using a box plot. Differential expression analysis between the two groups was conducted using the limma package, with |log FC| > 0.1 and *P* < 0.05 as the threshold criteria. These DEGs were then intersected with cuproptosis-related genes to identify cuproptosis-related DEGs [38]. By using the String database (https://string-db.org) [39] and Cytoscape 3.9.1 [40], a Protein-Protein Interaction (PPI) network was constructed to evaluate gene interactions (minimum required interaction score: medium confidence (0.400), all active interaction sources).

### 2.3 Identification of Candidate Cuproptosis-related Biomarkers

To predict the final cuproptosis-related biomarkers, this study applied two types of machine learning approaches. SVM, a widely utilized machine learning tool, was used for categorization and regression analysis. Additionally, we utilized the Recursive Feature Elimination (RFE) algorithm to screen the optimal genes from the metadata [41]. The LASSO analysis enhances prediction accuracy by regularizing a regressive analytical algorithm. LASSO regression was implemented in the R program using the glmnet package to determine the discriminative power of genes associated with ONFH and healthy specimens. Concurrently, the top 5 genes were identified using the Maximal Clique Centrality (MCC) algorithm based on the PPI network. The candidate cuproptosis-related biomarkers were determined by taking the intersection of the results from these three approaches.

The GSE74089 dataset from the GEO database was used to validate the expression of cuproptosis-related biomarkers. The ROC curve was utilized to assess the diagnostic value of these biomarkers. A logistics model was constructed using the R glm function in the pROC package [42], and the ROC was visualized using the ggplot2 tool.

### 2.4 Analysis of Causal Relationships through SMR

This study employed SMR, which utilizes summary-level data from GWAS and expression Quantitative Trait Loci (eQTL) studies to examine pleiotropic associations between gene expression levels and complex traits of interest. Additionally, the Heterogeneity in Dependent Instruments (HEIDI) test was applied, which uses all Single-Nucleotide Polymorphisms (SNPs) in cis-regulatory regions to differentiate between pleiotropic and linkage effects, thereby validating the causal associations between candidate cuproptosis-related biomarkers and ONFH [43]. The analysis of the SMR is based on the STROBE-MR checklist [44], and the parameters used are the default parameters. The eQTL data were obtained from the eQTLGen database (https://eqtlgen.org/) [45]. The GWAS data for the outcome used in the SMR analysis were sourced from the FinnGen database (https://www.finngen.fi/en; phenocode: OSTEON_DRUGS), which comprised 412181 cases (297 in the disease group and 411884 in the control group) [46].

### 2.5 Immune Infiltration Analysis

By using ImmuCellAI (http://bioinfo.life.hust.edu.cn/ImmuCellAI#!/analysis) [47], we identified the immunological responses of 24 distinct types of immune cells and evaluated the relationship between the candidate cuproptosis-related biomarkers and these immune cells using the R program.

### 2.6 LncRNA‒miRNA‒mRNA Network Prediction

Four databases, namely ENCORI, miRWalk, RNA22, and RNAInter, were used to predict the target miRNAs of candidate cuproptosis-related biomarkers [48-51]. Subsequently, validated lncRNAs were obtained from the HGNC database and intersected with the GSE123568 dataset by applying |logFC| ≥ 0.5 and adj. *p* < 0.05 as the threshold criteria to identify differentially expressed lncRNAs. Furthermore, the DIANA database [52] was employed to validate the interactions between these lncRNAs and the previously identified miRNAs.

### 2.7 Hypoxia/Ischemia(H/I) Model

Hypoxic conditions were established using a CO_2_ water-jacketed incubator (Baocheng, China), which can reduce O_2_ concentration to 0.5%. Ischemic conditions were induced by replacing the culture medium with glucose-free and serum-free DMEM (Gibco, USA). Bone Mesenchymal Stem Cells (BMSCs) were incubated in the CO_2_ water-jacketed incubator for 24 hours. Control samples were maintained under normoxic conditions for equivalent durations.

### 2.8 qRT‒PCR

mRNA: Total RNA was extracted from H/I-treated cells by using TRIzol reagent. cDNA was synthesized by reverse transcription at 65°C for 5 minutes, followed by incubation at 37°C for 15 minutes. qRT-PCR was performed with an initial denaturation at 95°C for 60 seconds, followed by 40 cycles of 95°C for 10 seconds and 60°C for 30 seconds. Additionally, a method for assessing relative expression levels was developed by utilizing ACTB (β-actin) as a reference gene for target gene normalization.

miRNA: miRNA was obtained, and cDNA was generated following the manufacturer’s protocol (Sangon Biotech). Standard samples were prepared according to the specified protocol. Subsequently, the samples were diluted, and qPCR was performed for mRNA analysis.

The relative RNA expression was quantified using the 2^-△△CT^ method. Table **1** displays the primers utilized in this investigation.

### 2.9 Statistical Analysis

Student’s t-test was performed to assess gene expression in diseased and healthy specimens. A *P*-value of < 0.05 was considered statistically significant. The core code pertaining to this study is available in Supplementary files **1**.

## 3 RESULTS

### 3.1 Identification of Cuproptosis-related DEGs

**Fig. (1):**
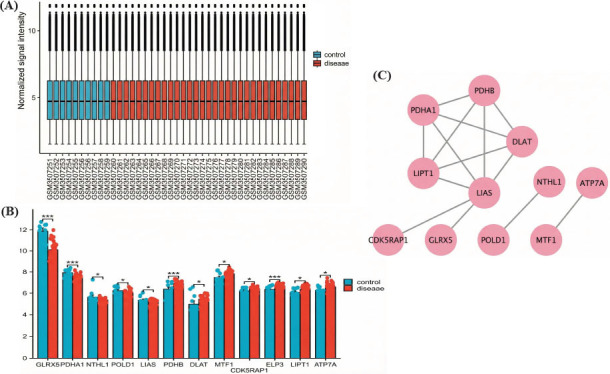
Identification of cuproptosis-related DEGs. (**A**) The box plot shows the normalization of the GSE123568 database. (**B**) The expression levels of 12 cuproptosis-related DEGs in the GSE123568 dataset (^∗∗∗∗^*p* < 0.0001, ^∗∗∗^*p* < 0.001, ^∗∗^*p* < 0.01, or ^∗^*p* < 0.05). (**C**) PPI network of cuproptosis-related DEGs.

The GSE123568 dataset was obtained from the GEO database and normalized (Fig. **1A**). Subsequently, differential expression analysis was conducted. Based on the results of this analysis, 12 cuproptosis-related DEGs were identified through the intersection of DEGs and cuproptosis-related genes. Among these, the genes *GLRX5, PDHA1, NTHL1, POLD1,* and *LIAS* were downregulated, while the genes *PDHB, DLAT, MTF1, ELP3, LIPT1, ATP7A,* and *CDK5RAP1* were upregulated in ONFH (Fig. **1B**). Additionally, a PPI network was used to describe the interactions between these genes, revealing connections among the proteins (Fig. **1C**). These findings suggest a potential link between ONFH and cuproptosis.

### 3.2 Identification of Candidate Cuproptosis-related Biomarkers

**Fig. (2):**
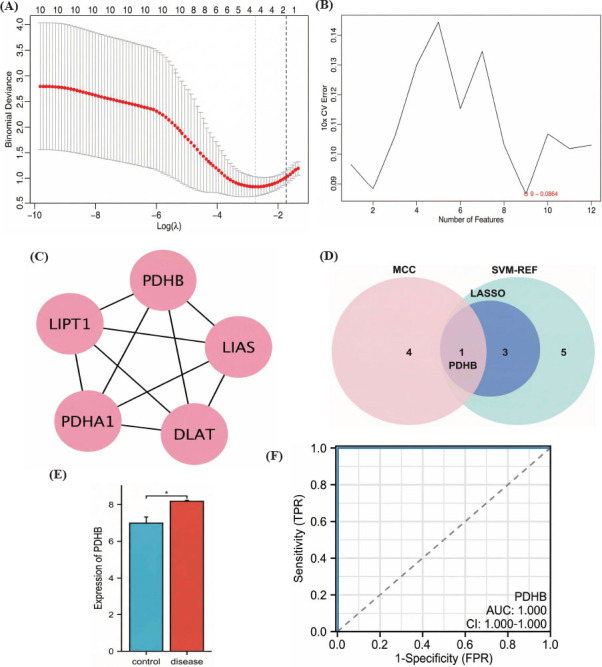
Identification of candidate cuproptosis-related biomarkers for ONFH. (**A**) Tuning feature screening in the LASSO algorithm. (**B**) A plot of cuproptosis-related biomarker screening through the SVM-RFE algorithm. (**C**) The top 5 genes were identified using the MCC algorithm. (**D**) *PDHB* is obtained by taking the intersection of the results of the LASSO, SVM-RFE, and MCC algorithms. (**E**) The expression of *PDHB* in the GSE74089 dataset (^∗^*p* < 0.05). (**F**) The ROC curve of *PDHB* in the GSE74089 dataset.

The 12 cuproptosis-related DEGs were further analyzed using the LASSO algorithm, and 4 cuproptosis-related biomarkers were obtained (Fig. **2A**). Concurrently, 9 cuproptosis-related biomarkers were identified *via* SVM-RFE (Fig. **2B**). Additionally, the top 5 genes were obtained using the MCC algorithm (Fig. **2C**). Subsequently, we identified one gene, *PDHB*, which was common across all three algorithms (Fig. **2D**). The GSE74089 dataset was utilized to validate the expression of *PDHB*; the results revealed its upregulation in the disease group (Fig. **2E**). The ROC curve analysis was then conducted to assess the diagnostic capability of *PDHB*, and the results demonstrated its efficacy as an individual diagnostic indicator (Fig. **2F**). However, due to the limitation of sample size, there may be overfitting. Furthermore, SMR results indicated that *PDHB* is a risk factor for ONFH (p_SMR = 0.029, b_SMR = 0.98) (Table **2**). These findings align with the previous analysis results. This suggested that the upregulation of *PDHB*, a cuproptosis-related biomarker, may contribute to the development of ONFH.

### 3.3 Immune Infiltration Analysis

**Fig. (3):**
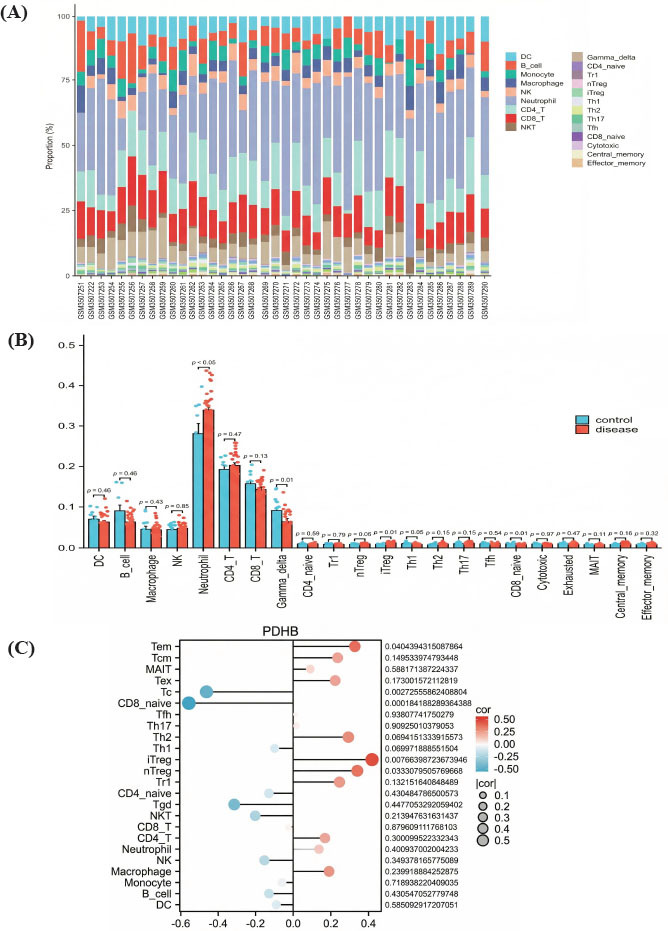
Immune infiltration analysis with ImmuCellAI. (**A**) Percentage of immune cells in the GSE123568 dataset. (**B**) Immune cell difference between the ONFH and control groups. (**C**) Correlation between *PDHB* and 24 types of immune cells.

Subsequently, we utilized ImmuCellAI to conduct immune infiltration analysis. The results revealed distinct distributions of immune cells in the ONFH group compared to the control group (Fig. **3A**). We also examined differences in immune features between the ONFH and control groups. Significant variations were observed in the levels of B cells, neutrophils, gamma delta T cells, iTreg cells, Th1 cells, and CD8 naive cells between the two groups (Fig. **3B**). Furthermore, the correlation between PDHB and immune cells in ONFH was analyzed. The analysis of immune cell ratios and *PDHB* expression demonstrated that *PDHB* was significantly associated with effector memory T cells, cytotoxic T cells, CD8 naive cells, iTreg cells, nTreg cells, and gamma delta T cells (Fig. **3C**). These findings suggest that *PDHB* likely plays a role in the pathogenesis and progression of ONFH by regulating immune cell infiltration.

### 3.4 lncRNA‒miRNA‒mRNA Network Prediction

**Fig. (4):**
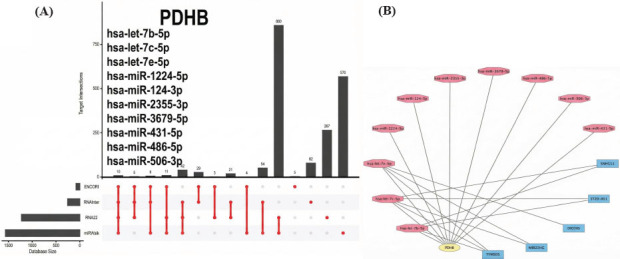
Prediction of the lncRNA‒miRNA‒mRNA network. (**A**) The miRNAs associated with *PDHB*. (**B**) The lncRNA‒miRNA‒mRNA network (mRNA, miRNA, and lncRNA are depicted in yellow, pink, and blue, respectively).

The miRNAs associated with PDHB were identified using the ENCORI, miRWalk, RNA22, and RNAInter4 databases (Fig. **4A**). Subsequently, 11 HGNC database-validated lncRNAs were identified in the GSE123568 dataset: *ASAP1-IT2, DIO3OS, LINC00482, LINC00570, LINC01013, MIR22HG, SNHG11, ST20, ST20-AS1, TMEM105*, and *TYMSOS*, with |logFC| ≥ 0.5 and adj. *p* < 0.05 as the threshold criteria. After validating the interaction between these 11 lncRNAs and miRNAs through DIANA, the lncRNA–miRNA–mRNA network was constructed (Fig. **4B**).

### 3.5 Validation of Expression of lncRNAs, miRNAs, and mRNAs

**Fig. (5):**
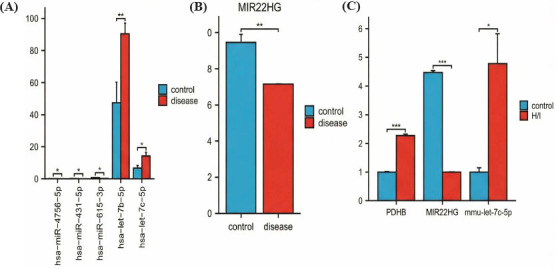
Validation of the expression of lncRNA, miRNAs, and mRNA. (**A**) Validation of miRNA expression in the GSE89587 dataset. (**B**) Validation of lncRNA expression in the GSE74089 dataset. (**C**) Validation of *PDHB, MIR22HG,* and *mmu-let-7c-5p* expression by qRT-PCR (^∗∗∗^*p* < 0.001, ^∗∗^*p* < 0.01, or ^∗^*p* < 0.05).

Following the validation of expression by using the GSE89587 dataset, five miRNAs with significantly differential expression were identified: *miR-4756-5p, miR-431-5p, miR-615-3p, let-7b-5p,* and *let-7c-5p* (Fig. **5A**). Furthermore, the validation of lncRNA expression using the GSE74089 dataset revealed that *MIR22HG* alone exhibited significant differential expression between the two groups (Fig. **5B**). Subsequently, qRT-PCR was used to verify the expression of these genes in the H/I model. The results demonstrated significant differential expression of *MIR22HG*, *let-7c-5p*, and *PDHB* between the two groups (Fig. **5C**). Based on these findings, we postulate a regulatory relationship between *MIR22HG*, *let-7c-5p*, and *PDHB*. Specifically, the downregulation of *MIR22HG* appears to induce the upregulation of let-7c-5p, which leads to the upregulation of PDHB and ultimately results in ONFH.

## 4 DISCUSSION

ONFH is a debilitating orthopedic condition that markedly affects patients’ quality of life. It is categorized as either traumatic or nontraumatic, with the majority of nontraumatic cases resulting from prolonged high-dose glucocorticoid use [53-55]. Recent studies indicate that various lncRNAs, miRNAs, and mRNAs play regulatory roles in the progression of ONFH [56-59]. Furthermore, emerging research has identified cuproptosis, a newly proposed form of cell death, as a contributing factor in several diseases, although its influence on ONFH remains unknown [14, 60-62].

In the present study, we utilized SVM-REF, LASSO regression analysis, and MCC algorithms to identify a cuproptosis-related gene, *PDHB*, which plays a crucial role in ONFH. Additionally, we performed SMR analysis to explore the causal relationship between *PDHB* and ONFH. Subsequently, immune infiltration analysis and lncRNA–miRNA–mRNA network prediction were conducted. Finally, we validated the expression of these genes using the external datasets GSE89587 and GSE74089 and qRT-PCR. The results of this study showed that the upregulation of the cuproptosis-related biomarker *PDHB* could lead to ONFH. The study also revealed that immune cells play a critical role in ONFH, and *PDHB* is notably associated with various immune cells. The study identified the existence of the *MIR22HG/let-7c-5p/PDHB* regulatory pathway, which may play a crucial role in ONFH through cuproptosis.

*PDHB*, pyruvate dehydrogenase E1 subunit beta, is located in the mitochondrion and is involved in the formation of the pyruvate dehydrogenase complex. It can catalyze the conversion of glucose-derived pyruvate into acetyl-CoA. Additionally, the pyruvate dehydrogenase complex is an integral component of the TCA cycle [63-66]. Consequently, *PDHB* may contribute to the pathogenesis of ONFH by regulating the TCA cycle and potentially inducing cuproptosis. Despite its potential significance, *PDHB* has been rarely studied in the context of ONFH. Baochuang Qi *et al.* [22] demonstrated a significant upregulation of *PDHB* expression in steroid-induced ONFH, which is consistent with our findings. Our research indicates that *PDHB* upregulation can lead to ONFH, possibly triggering cuproptosis by disrupting glycolysis and the TCA cycle. To further elucidate this mechanism, we predicted the related lncRNA-miRNA-mRNA network; the findings revealed multiple regulatory relationships involving *PDHB*, a cuproptosis-related gene. Notably, we identified the *MIR22HG/let-7c-5p/PDHB* regulatory axis, wherein *MIR22HG* downregulation results in *let-7c-5p* upregulation, subsequently leading to *PDHB* upregulation and ultimately resulting in ONFH. Chanyuan Jin *et al.* [67] reported that *MIR22HG* promoted osteogenic differentiation of hBMSCs through the *PTEN/AKT* signaling pathway. Additionally, Hui Long *et al.* [68] demonstrated the involvement of *MIR22HG* in OA progression through the *MIR22HG/miR-9-3p/ADAMTS5* axis. However, there have been few investigations on *let-7c-5p* and ONFH. The traditional notion is that miRNAs have an inhibitory effect on target genes. However, our findings indicate that *let-7c-5p* has a positive effect on *PDHB*. This is counter to conventional wisdom. Min Xiao *et al.* proposed NamiRNA, a new way of looking at miRNAs, in 2017. NamiRNA, a miRNA, differs from the miRNAs that are commonly known to inhibit gene expression. It can activate target genes by interacting with enhancers [69]. Many studies have shown that miRNAs can have a promoting effect on target genes [70-72]. Although our study suggests that the *MIR22HG/let-7c-5p/PDHB* axis triggers cellular cuproptosis leading to ONFH, further validation is necessary to confirm these findings.

Several recent studies have demonstrated the pivotal role of immune cell infiltration in ONFH [73-76]. We performed immune infiltration analysis and found substantial differences in various immune cells between the disease and control groups. Furthermore, *PDHB* was associated with multiple immune cells, including iTreg cells, CD8 naive cells, gamma delta T cells, and neutrophils. A recent study indicated that glucocorticoids can modulate immune cell inflammatory activation and anti-inflammatory polarization by enhancing the TCA cycle [77]. This suggests that glucocorticoids may regulate the TCA cycle by influencing the biological activity of *PDHB*, potentially triggering a local inflammatory response and ultimately leading to ONFH. Additionally, numerous studies have shown that T cell imbalance may promote ONFH [78-80], although the precise underlying mechanism remains unclear. Mayu *et al.* [81] observed that ONFH patients had neutrophils forming extracellular traps around the femoral head in small arteries. In summary, *PDHB* may contribute to ONFH by influencing the state of local immune cells; however, these mechanisms require further investigation.

This study has several limitations that warrant consideration. First, the datasets on ONFH were scarce, with the largest dataset comprising only 40 samples, which may introduce potential bias. At the same time, the SMR results may be biased due to the limitation of sample size. Future research should aim to validate these findings using a larger sample size. Second, the regulatory relationship of *MIR22HG/let-7c-5p/PDHB* was not experimentally validated in this study, necessitating further experimental analysis to elucidate this biological mechanism. Third, cuproptosis was not examined in the current study, and this aspect requires further investigation in subsequent research. Lastly, the limited sample size in the qRT-PCR analysis may restrict the generalizability of the results. Future studies should incorporate both *in vitro* and *in vivo* validation of the predicted regulatory mechanisms of *MIR22HG/let-7c-5p/PDHB* to provide a more comprehensive understanding of their clinical significance.

## CONCLUSION

This study identified *PDHB*, a biomarker associated with cuproptosis, whose upregulation can lead to ONFH development. Further investigation revealed that increased expression of the lncRNA *MIR22HG* can downregulate *PDHB* expression by downregulating the expression of the miRNA *let-7c-5p.* The downregulation of *PDHB* expression appears to play a crucial role in the etiology and progression of steroid-induced ONFH by modulating related immune cells. This research provides a novel perspective on the interrelationship between cuproptosis, immune infiltration, and ONFH. However, additional studies are necessary to validate these findings.

## Figures and Tables

**Table 1:** Specific sequences of the primers used in the qRT-PCR assay.

**Gene**	**Forward (5’-3’)**	**Reverse (5’-3’)**
*ACTB*	GGCAGCGGCAGGATACAC	TTCACAGGACACGAGCTG
*MIR22HG*	GTACATGGCTCTGCTGTCCTCATC	GTGCCTGCCTCTGTTGCTTCC
*PDHB*	CGCAAAGGTCCTAGAAGACAACTCC	ACACATCAGGTGAAGTCCCAACAAG
*mmu-let-7c-5p*	CCTGCTGGCTGTACAACCTTCTA	ATCCAGTGCAGGGTCCGAGG
	RT Primer: GTCGTATCCAGTGCAGGGTCCGAGGTATTCGCACTGGATACGACGGAAAG	

**Table 2:** Causal relationship between *PDHB* and ONFH (using SMR).

**Gene**	**b_SMR**	**se_SMR**	**p_SMR**	**p_HEIDI**
*PDHB*	0.98	0.45	0.029	0.45

## Data Availability

The datasets generated during and/or analysed during the current study are available in the GEO repository (https://www.ncbi.nlm.nih.gov/geo/), the FinnGen database (https://www.finngen.fi/en), and the eQTLGen database (https://eqtlgen.org/).

## LIST OF ABBREVIATIONS

BMSCs: Bone Mesenchymal Stem Cells
DEGs: Differentially Expressed Genes
eQTL: expression Quantitative Trait Loci
GEO: Gene Expression Omnibus
GWAS: Genome-Wide Association Studies
LASSO: Least-Absolute Shrinkage and Selection Operator
MR: Mendelian Randomization
ONFH: Osteonecrosis of the Femoral Head
PPI: Protein-Protein Interaction Network
qRT-PCR: Quantitative Real-time Polymerase Chain Reaction
ROC: Receiver Operating Characteristic
SMR: Summary statistics-based Mendelian Randomization
SNPs: Single Nucleotide Polymorphisms
SVM: Support Vector Machine
TCA: Tricarboxylic Acid

## STANDARDS OF REPORTING

STROBE-MR guidelines were followed.
